# Evaluating RGB channels in remote photoplethysmography: a comparative study with contact-based PPG

**DOI:** 10.3389/fphys.2023.1296277

**Published:** 2023-12-22

**Authors:** Rodrigo Castellano Ontiveros, Mohamed Elgendi, Giuseppe Missale, Carlo Menon

**Affiliations:** ^1^ Biomedical and Mobile Health Technology Lab, Department of Health Sciences and Technology, Zurich, Switzerland; ^2^ School of Electrical Engineering and Computer Science, KTH Royal Institute of Technology, Stockholm, Sweden; ^3^ Electronics and Telecommunications Department, Politecnico Di Torino, Torino, Italy

**Keywords:** rPPG, pulse oximentry, blood flow, volumetric changes, digital health, mobile health, non-contact assessment, remote monitoring

## Abstract

Remote photoplethysmography (rPPG) provides a non-contact method for measuring blood volume changes. In this study, we compared rPPG signals obtained from video cameras with traditional contact-based photoplethysmography (cPPG) to assess the effectiveness of different RGB channels in cardiac signal extraction. Our objective was to determine the most effective RGB channel for detecting blood volume changes and estimating heart rate. We employed dynamic time warping, Pearson’s correlation coefficient, root-mean-square error, and Beats-per-minute Difference to evaluate the performance of each RGB channel relative to cPPG. The results revealed that the green channel was superior, outperforming the blue and red channels in detecting volumetric changes and accurately estimating heart rate across various activities. We also observed that the reliability of RGB signals varied based on recording conditions and subject activity. This finding underscores the importance of understanding the performance nuances of RGB inputs, crucial for constructing rPPG signals in algorithms. Our study is significant in advancing rPPG research, offering insights that could benefit clinical applications by improving non-contact methods for blood volume assessment.

## 1 Introduction

Photoplethysmography (PPG) signals are instrumental in monitoring essential health indicators such as heart rate (HR) (Elgendi, 2020), heart rate variability (Mayampurath et al., 2018), blood pressure (Elgendi et al., 2019), atrial fibrillation (Pereira et al., 2020), and mental health (Lyzwinski et al., 2023). Recent advancements have facilitated the development of affordable smart wearables with contact PPG (cPPG) sensors for cardiac activity assessment (Elgendi, 2012; Mashhadi et al., 2015; Ayesha et al., 2021). A novel approach, remote PPG (rPPG), utilizes video cameras and RGB color channels for non-invasive cardiac signal extraction, aligning with the blood volume pressure (BVP) absorptivity spectrum (Zijlstra et al., 1991).

We hypothesize that the green channel is ideal for rPPG signal extraction. This is explored through algorithms like PCA (Lewandowska et al., 2011), CHROM-based rPPG (De Haan and Jeanne, 2013), LGI (Pilz et al., 2018), POS (Wang et al., 2016), and emerging deep learning methods (Yu et al., 2019; Schrumpf et al., 2021), over diverse datasets (Haugg et al., 2022; van Es et al., 2023). Understanding the reliability of RGB signals, influenced by recording settings and subject factors, is crucial when compared to cPPG for robust signal transformation (Charlton et al., 2023).

Previous research, including Verkruysse et al. (Verkruysse et al., 2008), Sinhal et al. (Sinhal et al., 2021), and Bhattacharjee et al. (Bhattacharjee and Yusuf, 2021), has explored the quality of rPPG signals from RGB data. While the green channel’s potential is acknowledged, the roles of blue and red channels, assessed under limited conditions, remain unclear. Our study addresses these limitations, utilizing three diverse datasets with varying camera types, pulse oximeters, lighting conditions, distances, and participant activities (Frey et al., 2022; Haugg et al., 2023).

We aim to identify the RGB channel in rPPG that most closely mirrors cPPG across different settings. Our objectives include assessing the similarity between RGB and cPPG signals in various contexts and statistically validating the robustness of the identified signal’s performance. This comprehensive analysis will enhance our understanding of cardiac insights and improve future video-based health monitoring techniques.

## 2 Materials and methods

### 2.1 Datasets

This study used three publicly-available datasets: LGI-PPGI, PURE and MR-NIRP. All three datasets include participants engaged in different activities while a video is recording them, and the cPPG signal is taken with a pulse oximeter.

**LGI-PPGI** (Pilz et al., 2018) is a dataset that contains videos from six participants, five males and one female. Each participant records a session doing four activities: Resting, Talking, exercising on a bicycle ergometer (Gym), and Rotation. The camera is a Logitech HD C270 webcam (25 fps), and the gold standard cPPG signals are taken with a pulse oximeter, CMS50E PPG device (sampling rate of 60 Hz). The camera video stream was captured uncompressed with autoexposure. The lighting condition depends on the activity; Talking is recorded outdoors, while the other activities are recorded indoors.

The **PURE** dataset (Stricker et al., 2014) consists of 10 participants, eight males and two females. They perform activities classified as Steady, Talking, Slow Translation, Fast Translation, Small Rotation, and Medium Rotation. An eco274CVGE camera by SVS-Vistek GmbH (30 fps) with a 640 × 480 pixel resolution and 4.8 mm lens was used. The pulse oximeter model is CMS50E (sampling rate of 60 Hz). The lighting is daylight through a window frontal to the face. The distance to the camera was 1.1 m, on average.

The **MR-NIRP** video dataset (indoor) (Magdalena Nowara et al., 2018) is composed of eight subjects, six males and two females, with different skin tones labelled by the data creator: one participant with an Asian skin tone, four with an Indian skin tone, and three with a Caucasian skin tone. The activities were Still and Motion, and in the latter, the participants talked and moved their heads. The main camera used is a FLIR Blackfly BFLY-U3-23S6C-C (sensor format ‘rggb’) with a resolution of 640 × 640 (30 fps). The device used to record the cPPG sequences was a CMS 50D + finger pulse oximeter (sampling rate of 60 Hz).

### 2.2 Data processing

**FIGURE 1:**
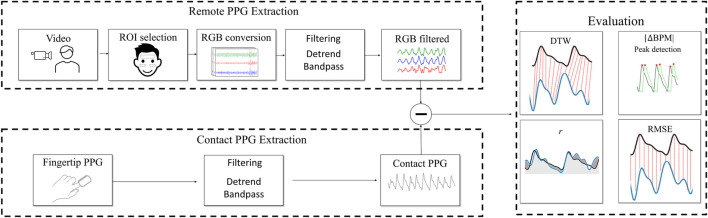
Visualization of the data extraction to remote photoplethysmograpm (rPPG) evaluation process. DTW = dynamic time warping, *r* = Pearson’s correlation coefficient, RMSE = root-mean-square error, |ΔBPM| = difference in heartbeats obtained from rPPG and contact PPG.

To obtain the rPPG signals that were later compared with the cPPG signals, several pre-processing steps were applied. A visualization is shown in Figure 1. The framework pyVHR (Boccignone et al., 2022) was implemented to do the preprocessing. For every video, the same procedure was applied. The regions of interest (ROIs) were extracted using the MediaPipe (Lugaresi et al., 2019) framework, which allowed the selection of 468 facial landmarks. Each landmark corresponds to a specific facial area, identified by a numerical label denoting its location. The choice was based on a combination of ROIs from the right cheek, left cheek, and forehead, as proposed by other authors (Kwon et al., 2015; Kim et al., 2021). Specifically, the landmarks were (107, 66, 69, 109, 10, 338, 299, 296, 336, 9) from the forehead, (118, 119, 100, 126, 209, 49, 129, 203, 205, 50) from the left cheek, and (347, 348, 329, 355, 429, 279, 358, 423, 425, 280) from the right cheek (Haugg et al., 2022). After extracting the ROIs, the average of each color was calculated for each frame. Consequently, each video consisted of a series of distinct frames, with each frame being characterized by three values denoting the average intensity across the Red, Green, and Blue (RGB) channels. This transformation effectively rendered the videos as signal representations, wherein each video was associated with three signals, each signal having a length equal to the number of frames within the video. Each signal is an rPPG signal, which will be compared to the cPPG signal extracted from the pulse oximeter.

To compare the ground truth cPPG and the rPPG, both signals were normalized according to robust normalization. This normalization is useful in cases where outliers exist, and it is more effective than other scaling techniques, such as min–max. The rPPG signals were downsampled to have the same sampling frequency as the cPPG signals, which allowed for comparison. Then, two filters were applied to the rPPG and cPPG: detrend and bandpass. The bandpass filter is a sixth order Butterworth filter with a cutoff frequency range of 0.65–4 Hz. The signals were divided into 10-s windows with no overlap, accounting for a total of 60 s per video. Therefore, the final dataset consisted of samples of 10 s of processed rPPG and the ground truth cPPG, which are compared with the metrics described later in this section.

### 2.3 Frequency domain

For the spectral analysis, Welch’s method was applied to every rPPG and cPPG window after preprocessing. The highest peak in the frequency domain was then chosen as the estimated HR. Other methods, such as autocorrelation, were implemented, but the absolute differences in |ΔBPM| were very small. We found this metric useful as it evaluates the frequency domain and it shows the capability of the rPPG signals from every channel to predict the HR.

### 2.4 Evaluation of the signals

Four metrics were used to evaluate the signals: dynamic time warping (DTW), Pearson’s *r* correlation coefficient, root-mean-square error (RMSE), and difference in heartbeats obtained from rPPG and cPPG (|ΔBPM|). For each video, the evaluation was done for every metric over 10-s windows. The resulting values were averaged to obtain the final results for each video. This enabled us to evaluate the results of every color channel from different perspectives.

#### 2.4.1 DTW

DTW (Müller, 2007) is an algorithm that measures the similarity between two time series, defined as:

Given two time series *A* = [*a*_1_, *a*_2_, … , *a*_*n*_] and *B* = [*b*_1_, *b*_2_, … , *b*_*m*_], where *n* and *m* are the lengths of the time series, the DTW distance between *A* and *B* is defined as:

$$\text{DTW}\left(A,B\right)=\min\left\{\overset{K}{\underset{k=1}{\sum }}d\left(i_{k},j_{k}\right)\right\}$$

where *K* is a warp path that maps the indices *i*_*k*_ of time series *A* to indices *j*_*k*_ of time series *B* and *d* (*i*, *j*) is a local distance function that measures the dissimilarity between data points *a*_*i*_ and *b*_*j*_.

It is particularly useful when the time series have different speeds and lengths. This applies to this case, given that, sometimes, the rPPG and its ground truth are not aligned, so other metrics that match timestamps are less suitable. The Python package DTAIDistance (Meert et al., 2020) was used to implement the metric.

#### 2.4.2 Pearson’s correlation coefficient (r)

The *r* coefficient measures the strength of the association between rPPG and cPPG using the following equation:

$$r=\frac{\sum_{i=1}^{N}\left(x_{i}-\hat{x}\right)\left(y_{i}-\hat{y}\right)}{\sqrt{\sum_{i=1}^{N}{\left(x_{i}-\hat{x}\right)}^{2}{\left(y_{i}-\hat{y}\right)}^{2}}}$$ (1)

where *x*_*i*_ and *y*_*i*_ are points at lag *i* of the rPPG and PPG signals, respectively. $\hat{x}$ and $\hat{y}$ represent their means. *N* is the number of points of the discrete signals.

#### 2.4.3 RMSE

RMSE is the standard deviation of the prediction error (i.e., residuals between the ground truth values and the extracted rPPG signals). It is expressed as follows:

$$RMSE=\sqrt{\frac{\sum_{i=1}^{N}{\left(x_{i}-y_{i}\right)}^{2}}{N}},$$ (2)

where *N* is the number of points and *x*_*i*_, *y*_*i*_ are the points at lag *i* of the rPPG and contact PPG signals, respectively.

#### 2.4.4 |ΔBPM|

Using Welch’s method, the HR was calculated with the power spectral density as the highest peak of the signal in the frequency domain. The range was restricted from 39 BPM to 240 BPM. To find the |ΔBPM|, the absolute difference in BPM from rPPG and the reference HR was calculated for every window, and then averaged.

## 3 Statistical tests

Statistical tests were conducted to compare the RGB channels. Non-parametric statistical tests are implemented, given that they do not make as many assumptions about the data as parametric statistical tests and, for some cases, the sample size is not large enough, e.g., comparing activities within a particular dataset where the number of subjects is small.

### 3.1 Friedman test

The Friedman test is suitable for this study because it compares the means of three or more groups. The following hypotheses were tested:

• Null hypothesis (H0): the medians of the groups are equal.
• Alternative hypothesis (H1): the median of at least one group is different.

The groups are represented by the three channels red, green, and blue. When RGB signals are compared among datasets or activities, the blocks are represented by the subjects. Within each block, the ranks are calculated (the idea of ranks is based on the order of the values, i.e., greater or less than) (Friedman, 1937). Then, for each group, find the average rank value as $R_{ij}=\frac{1}{n}\sum_{i=1}^{n}r_{ij}$ , where *i* represents the color channel, *i* = {1, … , *k*} and j represents the subject, *j* = {1, … , *n*}. The test statistic is approximated by: chi-squared distribution. It is expressed as

$$\chi_{F}^{2}=\frac{12n}{k\left(k+1\right)}\left[\overset{k}{\underset{j=1}{\sum }}R_{j}^{2}-\frac{k{\left(k+1\right)}^{2}}{4}\right].$$ (3)

If the *p*-value is significant, the means of the groups are equal, so the null hypothesis can be rejected. The next step is to use a *post hoc* test to calculate the pairwise group differences in the groups.

### 3.2 Nemenyi test

The Nemenyi test was applied to find the difference in the average ranking values and then to compare the difference with a critical distance (CD). The CD is defined as:

$$CD=q_{\alpha }\sqrt{\frac{k\left(k+1\right)}{6n}},$$ (4)

where *q*_*α*_ follow the Studentized range statistic divided by $\sqrt{2}$ (Demšar, 2006), and *α* = 0.05 for this study. If the difference for a given pair *R*_*i*_, *R*_*j*_, is greater than the CD, the difference is significant.

The general procedure is to apply the Friedmann test to each group (in our case it is the channels RGB) and if the *p*-value is significant, the difference in the means of the groups is different. In that case, the Nemenyi test is performed to rank the channels pairwise, i.e., green *versus* red, green *versus* blue and red *versus* blue.

## 4 Results

The main goal of the experiments was to show the differences between RGB channels in diverse settings. This was done by comparing the DTW, *r*, RMSE, and |ΔBPM| metrics. The first experiment was designed to evaluate the RGB signals across datasets. This makes it possible to assess whether the difference between the red, green, and blue channels is significant, depending on the dataset. For every dataset, there is a variation in the source of the illumination and the devices employed, including the camera and pulse oximeter. For the second experiment, the focal point was the activities conducted by the subjects. We analyzed the importance of the subjects’ activity and its impact on the performance of the rPPG signal. This makes it possible to differentiate the factors that can negatively alter the signal.

### 4.1 Analysis across datasets

**FIGURE 2:**
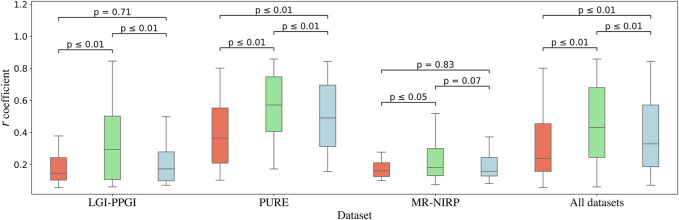
Similarity of each channel with the contact PPG, measured with the *r* coefficient. The results are shown for the datasets LGI-PPGI, PURE, and MR-NIRP. The *p*-value of every combination of channels, obtained with the Friedman test followed by the *post hoc* Nemenyi test, is represented by the bars above the boxplots.

Figure 2 shows the boxplots for every channel according to its *r* coefficient. As shown, for all the datasets, the green channel performs the best, followed by the blue and red channels, with *p*-values <0.01 . In contrast to the results for the combination of all datasets, LGI-PPGI and MR-NIRP showed no significant difference for red *versus* blue. Furthermore, the results for the PURE dataset agree with those for the combination of datasets and subjects. Indeed, the PURE dataset had the best performance in terms of the r coefficient, DTW, and RMSE (Table 1). The better the quality of the signal, the more evident the difference between the red, green, and blue channels. The *p*-values are shown in Table 2.

**TABLE 1:** Comparison of each color channel and the contact PPG (cPPG) across different activities and datasets. Results are averaged over subjects and time windows. The metrics DTW, *r*, and RMSE are shown. DTW = dynamic time warping, *r* = Pearson’s correlation coefficient, and RMSE = root-mean-square error.

		DTW	***r***	RMSE			DTW	***r***	RMSE
LGI-PPGI	Red vs. cPPG	39.66	0.19	3.14	Resting	Red vs. cPPG	7.38	0.37	1.19
	Green vs. cPPG	26.58	0.35	2.38		Green vs. cPPG	5.92	0.49	1.12
	Blue vs. cPPG	32.79	0.24	2.72		Blue vs. cPPG	6.81	0.41	1.16
PURE	Red vs. cPPG	8.66	0.39	1.29	Talking	Red vs. cPPG	12.61	0.2	1.46
	Green vs. cPPG	6.48	0.55	1.22		Green vs. cPPG	11.21	0.29	1.4
	Blue vs. cPPG	7.09	0.5	1.24		Blue vs. cPPG	11.95	0.25	1.43
MR-NIRP	Red vs. cPPG	9.97	0.19	1.24	Rotation	Red vs. cPPG	30.98	0.24	2.6
	Green vs. cPPG	7.64	0.23	1.08		Green vs. cPPG	17.96	0.45	1.9
	Blue vs. cPPG	9.29	0.2	1.19		Blue vs. cPPG	24.07	0.36	2.24
All datasets	Red vs. cPPG	15.97	0.32	1.71	Translation	Red vs. cPPG	6.86	0.51	1.24
	Green vs. cPPG	11.27	0.46	1.46		Green vs. cPPG	5.49	0.65	1.19
	Blue vs. cPPG	13.32	0.39	1.57		Blue vs. cPPG	5.86	0.61	1.21

**TABLE 2:** Comparison of the RGB channels regarding similarity to cPPG, with the purpose of finding significant differences between each channel. The *p*-values are shown for DTW, *r*, and RMSE for both experiments, obtained with the Friedman test followed by the *post hoc* Nemenyi test. The results for the datasets are shown on the left; the results for the participants’ activities are shown on the right.

		DTW	***r***	RMSE			DTW	***r***	RMSE
LGI-PPGI	Red vs. Green	0.001	0.001	0.293	Resting	Red vs. Green	0.001	0.001	0.001
	Red vs. Blue	0.365	0.712	0.293		Red vs. Blue	0.107	0.193	0.146
	Green vs. Blue	0.061	0.007	0.293		Green vs. Blue	0.001	0.001	0.146
PURE	Red vs. Green	0.001	0.001	0.001	Talking	Red vs. Green	0.001	0.028	0.007
	Red vs. Blue	0.001	0.001	0.002		Red vs. Blue	0.061	0.799	0.288
	Green vs. Blue	0.001	0.001	0.001		Green vs. Blue	0.018	0.123	0.288
MR-NIRP	Red vs. Green	0.001	0.017	0.001	Rotation	Red vs. Green	0.001	0.001	0.001
	Red vs. Blue	0.228	0.830	0.228		Red vs. Blue	0.007	0.003	0.023
	Green vs. Blue	0.003	0.073	0.003		Green vs. Blue	0.002	0.001	0.023
All datasets	Red vs. Green	0.001	0.001	0.001	Translation	Red vs. Green	0.001	0.001	0.001
	Red vs. Blue	0.001	0.001	0.001		Red vs. Blue	0.001	0.004	0.254
	Green vs. Blue	0.001	0.001	0.001		Green vs. Blue	0.140	0.004	0.001

### 4.2 Analysis across activities

The second experiment was designed to determine the differences between the RGB and cPPG signals based on the activity the subjects performed. This is also a useful way to detect whether RGB signals are prone to be unreliable when the subjects engage in different activities. The activities were divided into Resting, Talking, Rotation, and Translation. Rotation considers the Rotation activity from LGI-PPGI and the Small Rotation and Medium Rotation from the PURE dataset. Talking includes the Talking activity from the LGI-PPGI and PURE datasets and Motion from the MR-NIRP dataset (during the Motion activity, the subjects talk). Lastly, Translation includes the Small and Medium Translation activities from the PURE dataset.

The performance of the RGB colors is similar to the previous case, that is, the green channel is the best-performing signal, followed by the blue and red channels. Having said that, green always shows significantly better results than red, but this is not always the case with the green channel *versus* the blue channel. For the Rotation and Translation activities, the green signal outperformed the blue signal in terms of the r coefficient; however, in the other cases, the difference was not significant. The results for every metric and the *p*-values are listed in Tables 1 and 2. As shown, the results are consistent in terms of the RGB analysis of both the datasets and the activities.

### 4.3 HR and frequency domain

**FIGURE 3:**
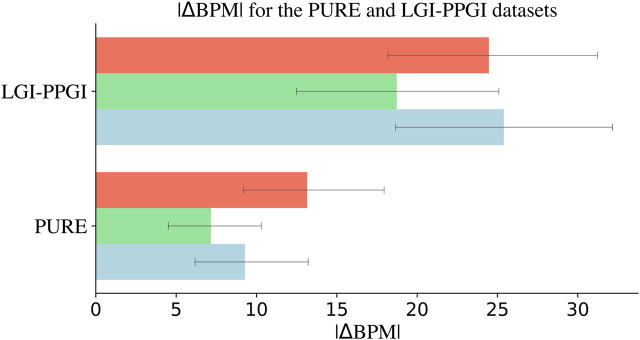
Heart rate estimation from each RGB channel with Welch’s method using the PURE and LGI-PPGI datasets, composed of different videos of 10 and 6 subjects, respectively, performing a wide range of activities. The blue signal shows better results than the red signal in PURE, but not in LGI-PPGI, where the blue signal, on average, is outperformed by the red signal. The green signal is consistently the optimal signal. Note that |ΔBPM| = absolute difference in heartbeats obtained from rPPG and contact PPG.

Welch’s method was used to quantify how each signal performs when predicting HR. A comparison of the HR for LGI-PPGI and PURE is shown in Figure 3. The estimation was done with the LGI-PPGI and PURE datasets because they were the only datasets that included the ground truth BPM. PURE is better than LGI-PPGI not only in terms of *r*, RMSE, and DTW, but also HR estimation. The results are shown in Table 3; the *p*-values are shown in Table 4. The green channel again outperformed the other channels, which followed the same pattern as the results of the morphology of the signals in the time domain. However, the red channel signal estimates the HR better than the blue channel signal in the LGI-PPGI dataset. It is important to note that the |ΔBPM| varies greatly between the PURE and LGI-PPGI datasets.

**TABLE 3:** Comparison of the heart rate estimated from each RGB channel and the ground truth heart rate. Results are shown for the |ΔBPM| metric for the comparison across activities and datasets. In both experiments and datasets, the green channel yields the most favorable outcomes. However, its variation shifts from 3.31 |ΔBPM| in Translation to 16.16 |ΔBPM| in Talking, showing great variation. Note that |ΔBPM| = absolute difference in heartbeats obtained from rPPG and contact PPG.

		|ΔBPM|			|ΔBPM|
LGI-PPGI	Red	24.47	Resting	Red	12.41
	Green	18.73		Green	7.33
	Blue	25.4		Blue	11.08
PURE	Red	13.15	Talking	Red	23.4
	Green	7.17		Green	16.16
	Blue	9.29		Blue	20.81
All datasets	Red	16.23	Rotation	Red	19.66
	Green	10.31		Green	11.61
	Blue	13.66		Blue	15.3
			Translation	Red	8.38
				Green	3.31
				Blue	3.99

**TABLE 4:** Comparison of the channels when estimating the heart rate against the ground truth to find significant differences between each channel. The *p*-values for |ΔBPM| are shown for both experiments, obtained with the Friedman test followed by the *post hoc* Nemenyi test. The results for the datasets are shown on the left; the results for the activities are shown on the right. Note that |ΔBPM| = absolute difference in heartbeats obtained from rPPG and contact PPG.

		|ΔBPM|			|ΔBPM|
LGI-PPGI	Red vs. Green	0.003	Resting	Red vs. Green	0.002
	Red vs. Blue	0.452		Red vs. Blue	0.280
	Green vs. Blue	0.088		Green vs. Blue	0.127
PURE	Red vs. Green	0.001	Talking	Red vs. Green	0.001
	Red vs. Blue	0.001		Red vs. Blue	0.061
	Green vs. Blue	0.027		Green vs. Blue	0.020
All datasets	Red vs. Green	0.001	Rotation	Red vs. Green	0.001
	Red vs. Blue	0.001		Red vs. Blue	0.004
	Green vs. Blue	0.003		Green vs. Blue	0.028
			Translation	Red vs. Green	0.001
				Red vs. Blue	0.002
				Green vs. Blue	0.556

**FIGURE 4:**
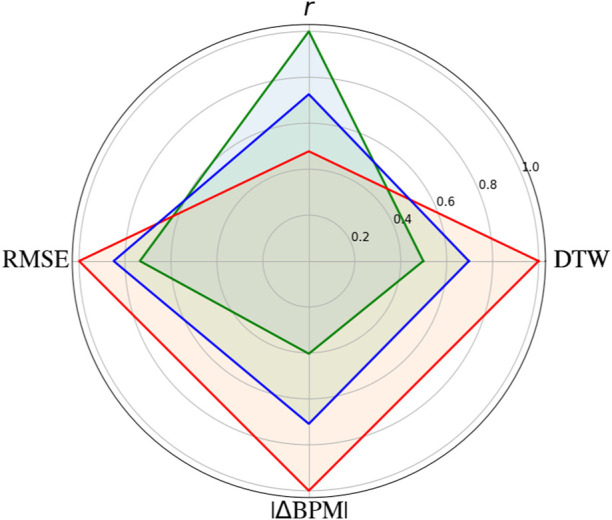
Overall performance for the red, green, and blue channels. The *r*, dynamic time warping (DTW), and root-mean-square error (RMSE) metrics were used. The results are shown for an average over subjects and time windows for each metric. The green channel consistently shows the best performance, followed by the blue and red channels. All the metrics are normalized to one.

To conduct an overall comparison of the RGB channels, all the metrics were visualized. The results are shown in Figure 4. Note that all the metrics are normalized to one. Overall, the green channel is best in terms of *r*, DTW, RMSE, and |ΔBPM|. In every case, the blue channel was ranked second and the red channel was ranked third.

## 5 Discussion

We assessed the performance of the RGB channels as rPPG signals for different datasets and activities, and we evaluated them using four metrics to rank the channels. First, the morphology of the signals was compared for *r*, RMSE, and DTW. For the comparison with datasets, the green channel showed better overall results than the blue and red channels. However, depending on the metric, some of the results are not statistically significant. This is the case of the red channel *versus* the blue channel for the MR-NIRP and LGI-PPGI datasets for all the metrics. Nonetheless, the PURE dataset results for every metric and channel were significant, with all of the *p*-values <0.01 and a better performance than the other datasets for every metric. This could be due to the quality of the signals and the settings in which the MR-NIRP and PURE datasets were obtained. Still, the green signal outperformed the red signal for every dataset. When the comparison is done across activities, the pattern is the same; the green channel is ranked first, followed by the blue channel and then the red channel. As the activity becomes more complex and involves more movements from the subject, the signal becomes unreliable, as can be the case for Rotation, with a DTW of 30.98 for the red channel in contrast to Resting, with a DTW of 5.92 for the green channel.

Regarding the spectral analysis, the results confirmed what had been previously shown in terms of the ranking of the channels. The |ΔBPM| was greater for the red signal, followed by the blue and green signals. Again, the results were better for the PURE dataset than for the LGI-PPGI dataset. This is partly due to the better morphology of the signals in the PURE dataset compared to the LGI-PPGI dataset. In some cases, the RGB signals are not reliable, especially when the settings in which the videos are recorded are not appropriate and the subject moves. For example, when the subject engages in fast rotation or is exercising on a bicycle in a gym, it can negatively affect the quality of the signal. Nevertheless, some algorithms, such as CHROM, perform well when abrupt changes in amplitude and shape occur in the signal due to different subject movements.

While other studies have confirmed these results, to date, there has been no exhaustive study of what channel is the best for different datasets, activities, and metrics. Most researchers used the green channel based on the results obtained by a research group in 2008 (Verkruysse et al., 2008). Still, that study (Verkruysse et al., 2008) aimed to demonstrate rPPG with ambient light, and the results were shown only for one participant at a time. This indicates that the green channel is a proper candidate, but there is no exhaustive scientific study that has confirmed it. One recent study (Sinhal et al., 2021) pointed out the difference between RGB signals and other algorithms, but only for one dataset, and it used fewer metrics. It is important to include several datasets because, as we have seen, the dataset has a significant impact on the results. Moreover, in that study (Sinhal et al., 2021), there was no analysis of how the subjects’ movements could affect the assessment. Another study (Lee et al., 2013), focused on the differences in RGB in relation to movement. That study had 12 subjects, and the movements were divided into horizontal and vertical. Our study included a wider range of movements and more subjects. They confirmed the results for HR but did not find statistically significant differences between green and blue for the SNR ratio (Lee et al., 2013).

We have shown that to obtain high-quality rPPG signals using only the RGB channels, the settings (such as the camera or illumination) in which the subjects’ activities are recorded is important because the variation in the inter-dataset is mostly due to those settings. Moreover, the cPPG signals of some datasets are obtained with unreliable devices, such as wristbands, instead of a pulse oximeter. Therefore, the rPPG from the RGB is not robust. Furthermore, although the RGB ranking results were the same, the values obtained for every metric showed differences between the activities. For the green channel, the |ΔBPM| obtained in the Resting activity was 7.73; it was 16.6 for the Talking activity. It is important to note that Resting has a higher |ΔBPM| than Translation because the latter only includes data from PURE, which is the best performing dataset. Overall, this leads to the conclusion that RGB signals should be recorded in situations where the subject is not moving and, more importantly, when the cameras and illumination conditions are adequate. While many modern algorithms overcome most of this reliability problem, when less noisy RGB signals with higher quality are obtained, the algorithms’ results are better.

## 6 Conclusion

In conclusion, the green channel signal exhibited the most favorable outcomes in all metrics assessed, followed by the blue and red channels. This conclusion was verified not only for signal morphology but also for HR estimation in the frequency domain. However, when analyzing the signal morphology, the RGB performance varied depending on the dataset and activity, with the selection of dataset having a significant impact on the rPPG for all metrics. The PURE dataset performed better than the LGI-PPGI and MR-NIRP datasets in terms of all metrics. Furthermore, the green channel provided the most accurate HR estimation in the frequency domain, with the blue and red channels following closely behind. Notably, for different activities, the |ΔBPM| exhibited a substantial change, ranging from 3.31 BPM for the green channel in the Resting activity to 33.9 BPM for the red channel in the Rotation activity. This implies that RGB signals are not resilient to diverse datasets and activities, which is an important consideration when utilizing them in clinical applications.

## Data Availability

Publicly available datasets were analyzed in this study. This data can be found here: The PURE dataset is available at https://www.tu-ilmenau.de/universitaet/fakultaeten/fakultaet-informatik-und-automatisierung/profil/institute-und-fachgebiete/institut-fuer-technische-informatik-und-ingenieurinformatik/fachgebiet-neuroinformatik-und-kognitive-robotik/data-sets-code/pulse-rate-detection-dataset-pure. The LGI-PPGI dataset is available at https://github.com/partofthestars/LGI-PPGI-DB. The MR-NIRP dataset is available at https://computationalimaging.rice.edu/mr-nirp-dataset/. The framework pyVHR can be downloaded from https://github.com/phuselab/pyVHR.
